# Multi-Omic Analysis of Symbiotic Bacteria Associated With *Aedes aegypti* Breeding Sites

**DOI:** 10.3389/fmicb.2021.703711

**Published:** 2021-08-12

**Authors:** Katherine D. Mosquera, Luis E. Martinez Villegas, Sacha J. Pidot, Chinhda Sharif, Sven Klimpel, Timothy P. Stinear, Luciano A. Moreira, Nicholas J. Tobias, Marcelo G. Lorenzo

**Affiliations:** ^1^Vector Behavior and Pathogen Interaction Group, Instituto René Rachou (FIOCRUZ), Belo Horizonte, Brazil; ^2^Mosquito Vectors: Endosymbionts and Pathogen-Vector Interactions Group, Instituto Ren Rachou (FIOCRUZ), Belo Horizonte, Brazil; ^3^Department of Microbiology and Immunology, The Doherty Institute, University of Melbourne, Melbourne, VIC, Australia; ^4^Institute for Ecology, Evolution and Diversity, Goethe University Frankfurt, Frankfurt, Germany; ^5^LOEWE Center for Translational Biodiversity Genomics (TBG), Frankfurt, Germany; ^6^Senckenberg Gesellschaft für Naturforschung, Frankfurt, Germany

**Keywords:** *Aedes*, *Klebsiella*, breeding sites, genomics, metabolomics

## Abstract

Mosquito breeding sites are complex aquatic environments with wide microbial diversity and physicochemical parameters that can change over time during the development of immature insect stages. Changes in biotic and abiotic conditions in water can alter life-history traits of adult mosquitos but this area remains understudied. Here, using microbial genomic and metabolomics analyses, we explored the metabolites associated with *Aedes aegypti* breeding sites as well as the potential contribution of *Klebsiella* sp., symbiotic bacteria highly associated with mosquitoes. We sought to address whether breeding sites have a signature metabolic profile and understand the metabolite contribution of the bacteria in the aquatic niches where *Ae. aegypti* larvae develop. An analysis of 32 mosquito-associated bacterial genomes, including *Klebsiella*, allowed us to identify gene clusters involved in primary metabolic pathways. From them, we inferred metabolites that could impact larval development (e.g., spermidine), as well as influence the quality assessment of a breeding site by a gravid female (e.g., putrescine), if produced by bacteria in the water. We also detected significant variance in metabolite presence profiles between water samples representing a decoupled oviposition event (oviposition by single females and manually deposited eggs) versus a control where no mosquito interactions occurred (PERMANOVA: *p* < 0.05; *R*^2^ = 24.64% and *R*^2^ = 30.07%). Five *Klebsiella* metabolites were exclusively linked to water samples where oviposition and development occurred. These data suggest metabolomics can be applied to identify compounds potentially used by female *Ae. aegypti* to evaluate the quality of a breeding site. Elucidating the physiological mechanisms by which the females could integrate these sensory cues while ovipositing constitutes a growing field of interest, which could benefit from a more depurated list of candidate molecules.

## Introduction

The ability of *Aedes aegypti* to thrive in urban environments and benefit from the available resources (i.e., humans as blood meal sources for reproduction and human-generated breeding sites for progeny), has made this mosquito a permanent threat to human health (Brady and Hay, 2020; Rose et al., 2020). Attempts to understand how this mosquito can effectively exploit nutrient-deficient artificial water-holding containers as breeding sites for its offspring to thrive have opened up new insights into mosquito bionomics (Dada et al., 2021).

The presence of diverse bacteria has been reported in breeding-site water collections, which are considered a fundamental component of larval niches (Wang et al., 2018; Scolari et al., 2019). This is because these immature forms rely on bacteria for their growth and development (Coon et al., 2014). Bacteria can be used as a food source and are often the most abundant microorganisms present in the larval diet (Rozeboom, 1935; Merritt et al., 1992). In general, breeding-site microbiota profiles seem to vary depending on mosquito species, aquatic habitat, and geographical origin (Muturi et al., 2018; Caragata et al., 2021; Juma et al., 2021). However, several bacterial isolates have been consistently detected in breeding sites, mosquito gut, and other mosquito-associated sources (Guégan et al., 2018). This is the case with *Klebsiella*, a genus of bacteria frequently reported in mosquitoes and their breeding sites (Gusmão et al., 2010; Chandel et al., 2013; Dada et al., 2014; Yadav et al., 2015; Wang et al., 2018; Hery et al., 2020; Alvarado et al., 2021; Rocha et al., 2021).

Several authors have identified *Klebsiella* associated with *Aedes*, *Anopheles*, and *Culex* mosquitoes using both culture-dependent and independent methods (Chandel et al., 2013; Yadav et al., 2015; Rocha et al., 2021). Therefore, it seems that these enteric bacteria maintain a stable relationship with the insects. For instance, *Klebsiella* has been isolated from different stages (eggs, larvae, pupae, and adults), tissues (midgut and ovaries), and sugar as well as blood-fed mosquitoes, both in laboratory and field populations (Gusmão et al., 2010; Chandel et al., 2013; Yadav et al., 2015; Wang et al., 2018; Alvarado et al., 2021; Rocha et al., 2021). Furthermore, Petri dishes filled with lyophilized *Klebsiella* resuspended in sterile water have proven to induce oviposition in *Culex pipiens* (Díaz-Nieto, 2014). This is particularly interesting considering that the water samples collected in natural breeding sites and domestic water storage containers have revealed the presence of *Klebsiella* (Dada et al., 2014; Hery et al., 2020; Rocha et al., 2021).

Oviposition-site selection by gravid females depends on water and container properties, with both biotic and abiotic factors shaping the decision on where to lay eggs (Hery et al., 2020). For instance, microbial communities present in water-holding containers have been shown to influence *Ae. aegypti* oviposition. Additionally, volatile compounds emitted by microorganisms act as attractants mediating egg-laying in a given container (Benzon and Apperson, 1988; Ponnusamy et al., 2008, 2015; Melo et al., 2020). Bacteria from breeding sites or water-soluble metabolites secreted by them have also been shown to impact abiotic factors of water, e.g., dissolved oxygen, and stimulate egg hatching by *Ae. aegypti* (Ponnusamy et al., 2011).

It has been suggested that gravid females transfer gut symbionts to aquatic niches where they oviposit (Coon et al., 2016). Indeed, this could disturb the pre-existing bacterial community of the water where the progeny will ultimately develop. In fact, midgut bacteria reported for larvae and adults have already been detected on the surface of *Ae. aegypti* eggs (Coon et al., 2014). It has been demonstrated that vertical transmission of microbes occurs mostly by egg-smearing, i.e., females impregnate the egg surface with symbiont-laden feces while ovipositing, allowing hatched larvae to acquire the symbionts (Salem et al., 2015; Sontowski and van Dam, 2020).

Microbial communities interact by producing metabolites that can influence the activity of neighboring microbes. These interactions can determine whether a microbe can subsist in an ecosystem and which features are desired to survive in a given community (Foster et al., 2017). In addition, microbially derived metabolites can also affect the host, helping with nutrition, protecting against pathogens, or causing disease (Fischer et al., 2017; Foster et al., 2017).

In order to investigate potential links between breeding sites and their resident microbes, we set out to address two specific questions: (i) what is the metabolic potential of bacteria commonly isolated in breeding sites? (ii) does the act of oviposition leave a metabolic fingerprint linking breeding sites and these bacteria?

## Materials and Methods

### Mosquito Rearing

*Aedes aegypti* (BR URCA strain, F5) were reared at 28 ± 2°C, 70 ± 10% relative humidity, and a 12:12 LL/DD photoperiod. Larvae were fed daily with half a tablet of Tetramin fish food, and pupae were transferred to cages before the adults emerged. Adults were offered 10% sucrose solution *ad libitum*, and one blood meal 7 days post-emergence using human blood. Human blood used to feed adult mosquitoes was obtained from a blood bank (Fundação Hemominas, Belo Horizonte, MG, Brazil), according to the terms of an agreement with Instituto René Rachou, Fiocruz/MG (OF.GPO/CCO agreement-Nr 224/16). Only fully engorged gravid females were used in the experiments 72 h after the blood meal.

### Bacteria Isolation, Culture Conditions, and Metabolite Extraction

Bacteria were isolated from fecal samples collected from gravid females. Briefly, individual females were placed inside sterile tubes containing 500 μl PBS for an hour. The feces were homogenized and a 25 μl aliquot of this homogenate was plated on MacConkey agar. Subsequently, the plates were incubated at 25°C for 24 h and sub-cultured on fresh plates to obtain pure single colonies. Colony morphology was inspected for shape, elevation, margin, texture, and pigmentation. DNA samples were extracted using the DNeasy Blood & Tissue Kit (Qiagen), according to the manufacturer’s manual. For taxonomic identification, the 16S rRNA gene was amplified by using the forward primer ENV1 and the reverse primer ENV2 and Sanger sequenced.

For metabolite extractions, bacteria were inoculated in TSB 1%, TSB 0.1%, TSB 0.01%, LB 1%, and LB 0.1%. We opted to switch media from MacConkey to provide a less restricting range of nutrients so that the bacteria could produce as broad a range of metabolites as possible. Furthermore, since breeding sites and their respective nutrients vary greatly in nature, we sought simply to obtain an overview of what *Klebsiella* can produce and whether this correlates with our experimental breeding site setup. The cultures were incubated at 25°C and 200 rpm up to 36 h. For each medium concentration, 500 μl of bacterial culture were mixed with 500 μl of methanol. The mixtures were vortexed and centrifuged for 30 min at 20,000 rcf. 50 μl of the supernatants were recovered and concentrated in a speed vac.

### Genome Sequencing, Assembly, and Analysis

Bacterial genomic DNA was extracted as described in section “Bacteria Isolation, Culture Conditions, and Metabolite Extraction.” Isolated DNA was sequenced on the Illumina NextSeq 500 platform. DNA libraries were constructed using the Nextera XT DNA preparation kit (Illumina) and whole-genome sequencing was performed using 2 × 150 bp paired-end chemistry. A sequencing depth of >50× was targeted for each sample.

Raw sequencing reads were trimmed using trimmomatic (v0.38). Sequences were then assembled using SPAdes (v3.12.0) (Bankevich et al., 2012). *Klebsiella* contigs along with several other genomes (Supplementary Table 1) were submitted to gutSMASH (v1.0.0) to identify specialized primary metabolite pathways.

### Breeding Site Metabolite Extraction and Analysis

**FIGURE 1:**
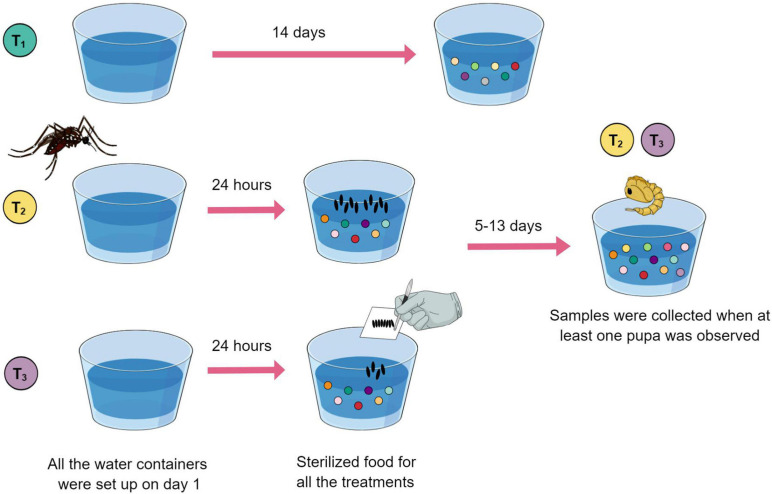
Eleven breeding sites and five controls were set up using a plastic cup filled with type I water (day 1) plus sterilized food solution (day 2). Treatment 1 (green) cups served as experimental controls. Treatment 2 (yellow) breeding sites were visited by an ovipositing female, individually. Treatment 3 (purple) breeding sites were set up with manually deposited eggs. Controls were collected on day 14. Breeding site water samples that had larvae developing were collected when at least one pupa was observed.

To investigate the relationship between bacterial metabolites present in *Ae. aegypti* breeding sites and oviposition, two types of breeding sites, and one control condition were defined for comparison (Figure 1). Breeding sites and control containers were set up separately inside mesh cages presenting one plastic cup filled with 80 ml of type I water and 500 μl of sterilized food solution. Five replicate samples containing water plus food served as experimental controls, i.e., no mosquitoes visited them. Additionally, six breeding sites visited by a single gravid female were generated separately. For this, mosquitoes were allowed to lay eggs for 24 h and then removed. Subsequently, the number of eggs laid in each cup was counted using a magnifying glass. This allowed us to calculate the average amount of eggs needed for preparing breeding sites by manually depositing eggs, avoiding the direct action of the female. Another set of five breeding sites was set up with manually deposited eggs. These eggs were derived from groups of gravid females allowed to oviposit on pieces of humid filter paper, which were dried and stored (a standard egg collection method for rearing mosquitoes in insectary conditions; see Imam et al., 2014).

Water samples from controls were collected on day 14. Water samples that had larvae developing were collected when at least one pupa was observed (between 6 and 14 days after the experiment was initiated). Larvae and pupae were removed, and the water samples were frozen and lyophilized.

Dried extracts were then resuspended in 1 ml of methanol, centrifuged for 30 min at 13,000 rpm. Liquid chromatography-mass spectrometry (LC-MS) analysis was performed on a Thermo Scientific UltiMate 3000 System using a C18 column (ACQUITY UPLC BEH C18 Column, 1.7 μm, 2.1 mm X 50 mm, Waters) linked to a Bruker Impact II System (Bruker Daltonik GmbH). Runs were performed using a gradient of MeCN/0.1% formic acid in H2O (5:95% to 95:5% over 16 min). Data acquisition was performed as previously described (Tobias et al., 2017). Acetonitrile was used as a control for blank measurements.

### Network Analysis Details

The relevance of isolating extracted metabolites from bacterial cultures and treatment samples was to be able to pinpoint if the metabolites extracted from the water had a bacterial origin (either *Klebsiella* or other bacteria with similar metabolic pathways capable of the same chemical output). A molecular network was created with the Feature-Based Molecular Networking (FBMN) workflow (Nothias et al., 2020) on the GNPS platform (Wang, 2016). The mass spectrometry data were first processed with MZMINE2 (Pluskal et al., 2010) and the results were exported to GNPS for FBMN analysis. The data were filtered by removing all MS/MS fragment ions within ±17 Da of the precursor m/z. MS/MS spectra were window filtered by choosing only the top 6 fragment ions in the ±50 Da window throughout the spectrum. The precursor ion mass tolerance was set to 0.2 Da and the MS/MS fragment ion tolerance to 0.2 Da. A molecular network was then created where edges were filtered to have a cosine score above 0.7 and more than 6 matched peaks. Further, edges between two nodes were kept in the network if and only if each of the nodes appeared in each others respective top 10 most similar nodes. Finally, the maximum size of a molecular family was set to 100, and the lowest scoring edges were removed from molecular families until the molecular family size was below this threshold. The analog search mode was used by searching against MS/MS spectra with a maximum difference of 100.0 in the precursor ion value. The library spectra were filtered in the same manner as the input data. All matches kept between network spectra and library spectra were required to have a score above 0.7 and at least 6 matched peaks. The DEREPLICATOR was used to annotate MS/MS spectra (Mohimani et al., 2018). The molecular networks were visualized using Cytoscape (v3.5.1).

### Statistical Analyses

To address if the interactions between gravid females and developing larvae, with the community of microorganisms in the breeding sites were reflected in the chemical composition of the samples at the time of collection, we filtered the detected metabolites table (Supplementary Table 2) transforming it into a presence and absence data set. This binary matrix was then utilized to estimate the Jaccard dissimilarities between treatments, visualize the constrained ordination patterns based on the sample profiles and their group affiliation, and evaluate if the differences between each treatment were significant by means of a pairwise PERMANOVA. All the analyses were performed in the Rstudio environment v1.1.423 (R Core Team, 2020) using the following packages: vegan v2.5-7 (Oksanen et al., 2020), mctoolsr v0.1.1.2, and pairwiseAdonis v0.0.1 (Martinez Arbizu, 2020).

## Results and Discussion

### Isolation, Identification, and Description of *Klebsiella* sp. MC1F From *Aedes aegypti* Feces

Circular, convex, regular margined, mucoid, and pink colonies were recovered from MacConkey agar plates. After DNA isolation, 16S rRNA amplicon, and genome sequencing, the bacterium was identified as *Klebsiella* sp. Indeed, this bacterial taxon has often been isolated from our mosquito strain (BR URCA, F5). Assembly of *Klebsiella* reads resulted in 175 contigs (N50: 167,915 bp) totaling 5,305,341 bp and a GC content of 57.9%. The genome is available under accession number JAGTYC000000000.

Several authors have reported the association between *Klebsiella* and mosquitos, including its detection on eggs, larvae, pupae, adults, and their breeding sites (Gusmão et al., 2010; Chandel et al., 2013; Dada et al., 2014; Yadav et al., 2015; Wang et al., 2018; Hery et al., 2020; Rocha et al., 2021). Culture-dependent approaches have allowed the isolation of *Klebsiella* from the midgut of *Ae. aegypti* adults emerging from larvae and pupae collected from natural breeding sites (Yadav et al., 2015) and from domestic water storage containers (Dada et al., 2014). Using high-throughput 16S rRNA amplicon sequencing, *Klebsiella* has been reported in natural aquatic habitats and in the *Ae. aegypti* larvae developing there (Hery et al., 2020). Díaz-Nieto et al., 2016 have isolated *Klebsiella* from all stages of *Culex pipiens* (for isolation from mosquito gut also Díaz-Nieto, 2014). This bacterial taxon has also been identified in all developmental stages of *Anopheles darlingi* and their breeding environments suggesting a narrow relation between these bacteria and their hosts (Rocha et al., 2021). In addition, our pilot shotgun metagenomic sequencing studies from *Aedes japonicus* larvae and breeding site water (collected from a water basin in a cemetery in Wiesbaden, Germany, coordinates: 50.104510, 8.216475), has identified Gammaproteobacteria, which includes *Klebsiella*, constituting 5 and 9% of all bacterial reads, respectively (Supplementary Figure 1). Based on these consistent findings, we suggest that *Klebsiella* is a potential mosquito symbiont that can be found in all stages and transferred to water by females to support early larval development in breeding sites.

Gut bacterial communities can be vertically transferred from adults to eggs as evidenced for tephritid fruit flies and *Ae. aegypti* (Lauzon et al., 2009; Coon et al., 2014). These bacterial taxa detected on insect eggs were also found in the gastrointestinal tracts of adults. The mechanisms by which this transference would occur are not yet elucidated, but one route of interest to our work involves adults transmitting symbionts by smearing fecal matter on the eggshells during oviposition (Salem et al., 2015; Sontowski and van Dam, 2020). In the case of mosquitoes, this simple process would allow the larvae to acquire the bacteria shortly after hatching or even use them for their initial nurture (Díaz-Nieto et al., 2016). Indeed, throughout the execution of our experiments, we observed that gravid females eventually defecated in breeding sites. As bacterial transference through such a mechanism has been reported in other insect models (Engel and Moran, 2013), we suggest that future studies should test whether this is a stereotypical behavior exhibited by egg-laying females or a stochastic event.

Egg-laying decisions by female *Ae. aegypti* are crucial to grant offspring survival and optimal development. Bacteria belonging to the genus *Klebsiella* have been shown to mediate attraction and induce oviposition in *Cx. pipiens* gravid females (Díaz-Nieto et al., 2016). Moreover, *Klebsiella* nurtures the most vulnerable stage of the mosquito life cycle, L1, to ensure molting to a more resilient stage, L2, and thus increase the larvae’s chances of reaching adulthood (Díaz-Nieto et al., 2016). This could be particularly important for *Ae. aegypti* considering its ability to exploit small and temporary rainwater collections as larval breeding sites (Scolari et al., 2019).

### Metabolic Pathways Present in *Klebsiella* sp. MC1F

**FIGURE 2:**
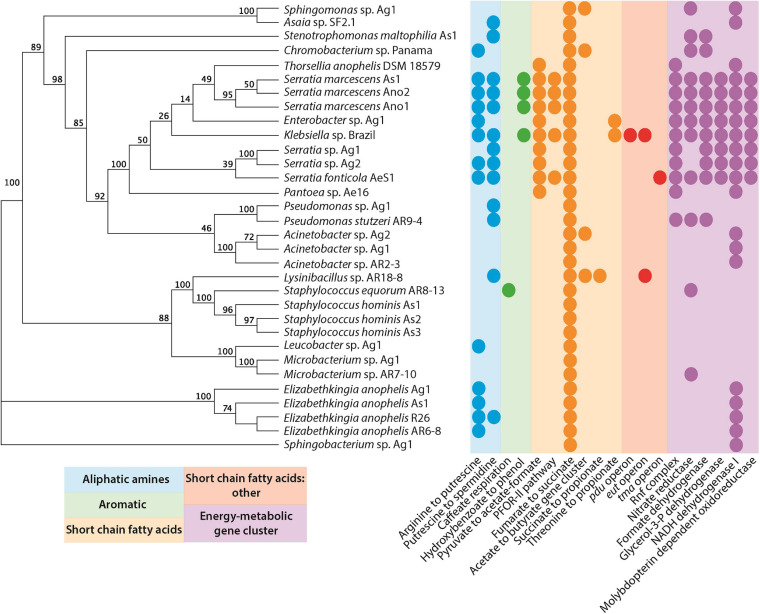
Primary metabolic pathways present in bacteria isolated from mosquitoes (Supplementary Table 1). Genomes were submitted to gutsmash (https://gutsmash.bioinformatics.nl/) to identify metabolic pathways related to the production of aliphatic amines, aromatic compounds, short-chain fatty acids, or energy metabolic clusters. Circles represent the presence of at least a single copy of the labeled pathway. The tree represents a maximum likelihood phylogeny based upon the 16S rRNA sequence with 100 bootstraps.

Several studies have shown that microbes are indispensable for mosquito larval development (Coon et al., 2014; Wang et al., 2018). Given the importance of bacteria in breeding sites, we wanted to investigate the metabolic pathways present in organisms that have been isolated from multiple mosquito sources. We concur with the hypothesis that a number of key microbial species present in breeding sites might be deposited by the mosquitoes themselves. We used a recently developed tool, gutSMASH (Andreu et al., 2021), to analyze primary metabolic pathways present in the genomes of facultatively anaerobic as well as aerobic bacteria that have been isolated from mosquitoes (Ganley et al., 2020; Figure 2 and Supplementary Table 1).

Only a single pathway is present in all of the bacteria examined: the fumarate to succinate pathway. This is perhaps unsurprising since the conversion of fumarate to succinate, mediated by the fumarate reductase enzyme, is an important part of the anaerobic respiration pathway in microbial metabolism (Lu and Imlay, 2017). A number of other short-chain fatty acid pathways are also present, none of which are ubiquitous to all species. However, the pyruvate to acetate/formate pathway was widespread in the Gammaproteobacteria. The pyruvate formate lyase enzyme mediates this reaction, and helps regulate anaerobic glucose metabolism. However, the enzyme is also involved in two other metabolic pathways: butanoate metabolism and propanoate metabolism. Interestingly, butanoate and propanoate esters actually display mosquito repellent effects in *Anopheles stephensi*, perhaps pointing to a role for bacteria in mediating the oviposition site selection by gravid mosquitoes (Sharma et al., 2009). Further to this, the presence of aliphatic amine and aromatic pathways may additionally support this role of some bacteria. For instance, *Ae. aegypti* mosquitoes are attracted to oviposition sites baited with putrescine (Hussain et al., 2016). Moreover, high levels of putrescine and spermidine have been detected in *Ae. aegypti* ovaries. It has been suggested that these polyamines could be stored in eggs for use during embryogenesis (Kogan and Hagedorn, 2000). This is particularly interesting considering that *Klebsiella* has been isolated from *Ae. aegypti* ovaries (Alvarado et al., 2021). Polyamines have been implicated in growth processes and were highlighted as one of the most important metabolites produced by the intestinal microbiota that affect host health and disease (Matsumoto et al., 2011). A metabolomic study in mice demonstrated that colonic microbiota is primarily responsible for the intestinal luminal concentrations of putrescine and spermidine (Matsumoto et al., 2012). In *Drosophila melanogaster*, a diet high in polyamines was shown to be beneficial and increased its reproductive success (Hussain et al., 2016). Furthermore, the exogenous addition of polyamines, especially spermidine, extended the lifespan in yeast, nematodes, and flies (Eisenberg et al., 2009; Morselli et al., 2009). Therefore, the presence of bacteria producing these metabolites in mosquito breeding sites may play a key role in promoting the successful development of larvae and the fitness of adults.

It is important to highlight that metabolites from other pathways could also influence the female-perceived quality of mosquito breeding sites. Short-chain fatty acids, like isovaleric acid and butyric acid, have been shown to act as deterrents to ovipositing females of *Culex* spp. and *Aedes albopictus* (Hwang et al., 1980; Boullis et al., 2021). Likewise, aromatic compounds like indole mediate oviposition of *Anopheles gambiae* and *Culex* spp. (Millar et al., 1992; Blackwell and Johnson, 2000; Lindh et al., 2008).

### Bacterial Metabolites Are Present in Breeding Sites Visited by Female Mosquitoes

One of our main objectives was to describe and measure the effects that oviposition and larval development have on the metabolite composition of breeding sites. To do so we separated the two main interactions mosquitoes have with the water in the breeding site: the direct contact of females with water (and any stereotypical behavior this entails, e.g., grooming, tasting, and defecating, etc.), and the contact of eggs, their eclosion, and larval development. We sought to compare whether water samples exposed to each of these conditions presented qualitatively dissimilar metabolite profiles, i.e., presence/absence. As it can be observed in the RDA plot (Supplementary Figure 2), the clustering patterns suggest the biotic and abiotic conditions represented in each treatment generated distinct metabolite profiles at the endpoint of the experiment. Samples from each treatment distributed along the ordination axes in a well-resolved pattern. The constrained variance in the reported plot amounted to 23.89%. Furthermore, the pairwise PERMANOVA revealed that the dissimilarities among treatments were all significant (Supplementary Table 3). The effect size (R^2^) of the conditions represented by each treatment when compared were as follows: Treatment 1vs Treatment 2 24.64%; Treatment 1vs Treatment 3 30.07%; and Treatment 2vs Treatment 3 21.20%. The remaining variance could be due to relevant interactions between biotic and abiotic explanatory variables that change throughout time and larval development (e.g., bacterial community composition and their metabolic output in response to changes in pH, dissolved oxygen, and conductivity, etc.). Physicochemical parameters were recently described as relevant features of *Ae. aegypti* breeding sites (Hery et al., 2020) and their fluctuations may be drivers (or responses) of bacterial community structures, and thus of their individual and community metabolism. We suggest that the layer of information generated by metabolomic assays may prove relevant as it provides a new source of analytical signal to gain insights into this dimension of the niche where larvae develop and shape key life-history traits (Dickson et al., 2017). For instance, this approach could be enhanced by interrogating similar sample types using other solvents, as methanol may not extract the entire breadth of metabolites present.

**FIGURE 3:**
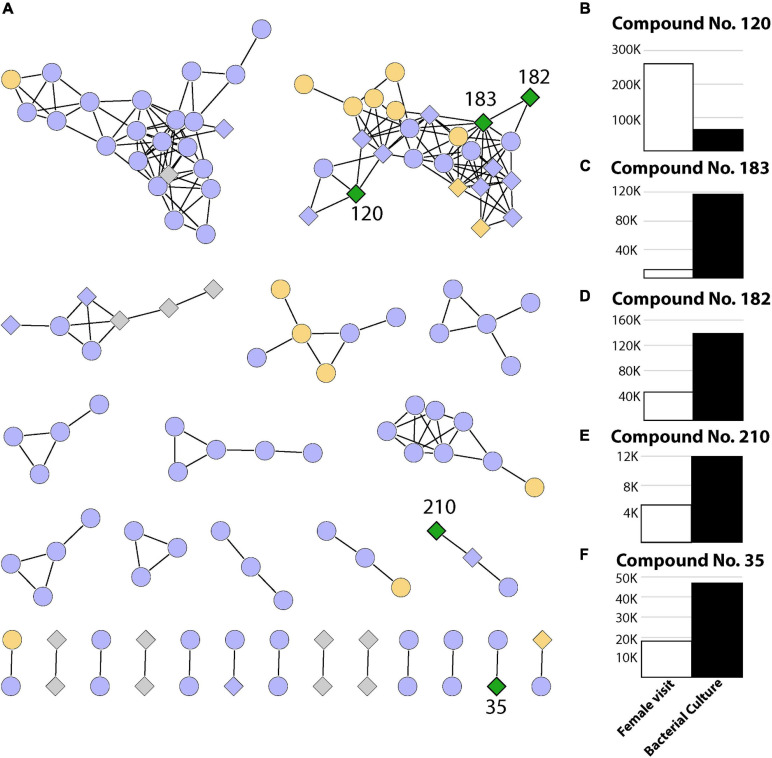
Summary of 239 metabolites identified in this study. **(A)** Networks containing two or more metabolites (nodes) connected by edges when a minimum number of matching fragments is present (see section “Materials and Methods”). The coloring represents metabolites identified in experiments where only the female visited (T_2_, green), where the female visited and/or eggs were manually placed (T_2_ and/or T_3_, orange), where metabolites were present in the control sample and/or T_3_ and/or T_2_ (purple), as well as metabolites detected only in bacterial cultures (gray). Diamonds represent bacterial metabolites, while circles represent metabolites not present in bacterial samples. **(B–F)** Relative abundance (area under the curve from mass spectrometry) for compounds present in breeding site experiments where the female visited (T_2_) and also present in bacterial extracts (green diamonds).

Finally, as we can observe in Figure 3A, there were five metabolites that had both a bacterial origin and were present in T_2_ which represents the act of oviposition and larval development closer than it occurs in nature (Figures 3B–F). These metabolites were absent from both the control (T_1_) and manual oviposition conditions (T_3_), therefore representing discriminative features among the chemical composition of the samples representing a decoupled oviposition. As these metabolites are unique to the combination of biotic variables, they could potentially act as indicators of successful female oviposition, egg eclosion, and larval development in *Ae. aegypti*. It is relevant to highlight that these metabolites could also originate from bacteria within the breeding site pertaining to taxa with which *Klebsiella* shares metabolic pathways. This is plausible as functional redundancy can be expected in microbial systems (Louca et al., 2018).

## Future Directions

Metabolites of bacterial origin, particularly from *Klebsiella* (or other related taxa with similar metabolic outputs) could act as guiding and/or decision-making modulating sensory cues (either olfactory or gustatory) for other conspecific females to access the quality of the breeding site (Ponnusamy et al., 2008; Boullis et al., 2021 and references therein). It is known that *Klebsiella* is present in larvae-positive breeding sites (Dada et al., 2014) and that bacteria from this genus produce volatile compounds (Rees et al., 2017). As thriving larvae alter their niche, potentially modifying the microbial composition (Scolari et al., 2021), these bacteria-derived volatile compounds could accumulate in the headspace of the water. Identifying the nature of these molecules, and using them in chemical ecology and behavioral assays seems to be a promising avenue to explore, as Boullis et al. (2021) recently highlighted, due to the fact that their mechanism of action has not yet been elucidated.

## Data Availability Statement

The *Klebsiella* sp. MC1F assembly and annotation can be found in Genbank with accession number JAGTYC000000000. All metabolomic data are freely available at the MassIVE repository under accession number MSV000087341, ftp://massive.ucsd.edu/MSV000087341/.

## Author Contributions

KM, LEM, CS, SP, SK, TS, LAM, NT, and ML conceived and designed the analysis. KM, LEM, SP, CS, and NT collected the data. KM, LEM, SP, CS, SK, TS, NT, and ML contributed to data or analysis tools. KM, LEM, SP, CS, LAM, NT, and ML performed the analysis. KM, LEM, NT, and ML wrote the initial draft. All authors read and approved the final draft.

## Conflict of Interest

The authors declare that the research was conducted in the absence of any commercial or financial relationships that could be construed as a potential conflict of interest.

## Publisher’s Note

All claims expressed in this article are solely those of the authors and do not necessarily represent those of their affiliated organizations, or those of the publisher, the editors and the reviewers. Any product that may be evaluated in this article, or claim that may be made by its manufacturer, is not guaranteed or endorsed by the publisher.
