# Supplementary material for: Multi-Omic Analysis of Symbiotic Bacteria Associated With Aedes aegypti Breeding Sites
Source: Front Microbiol. 2021 Aug 12;12:703711. doi: 10.3389/fmicb.2021.703711 (PMC8406634; doi:10.3389/fmicb.2021.703711)
Supplement: Supplementary file 1 [file Data_Sheet_1.pdf]

Supplementary Material

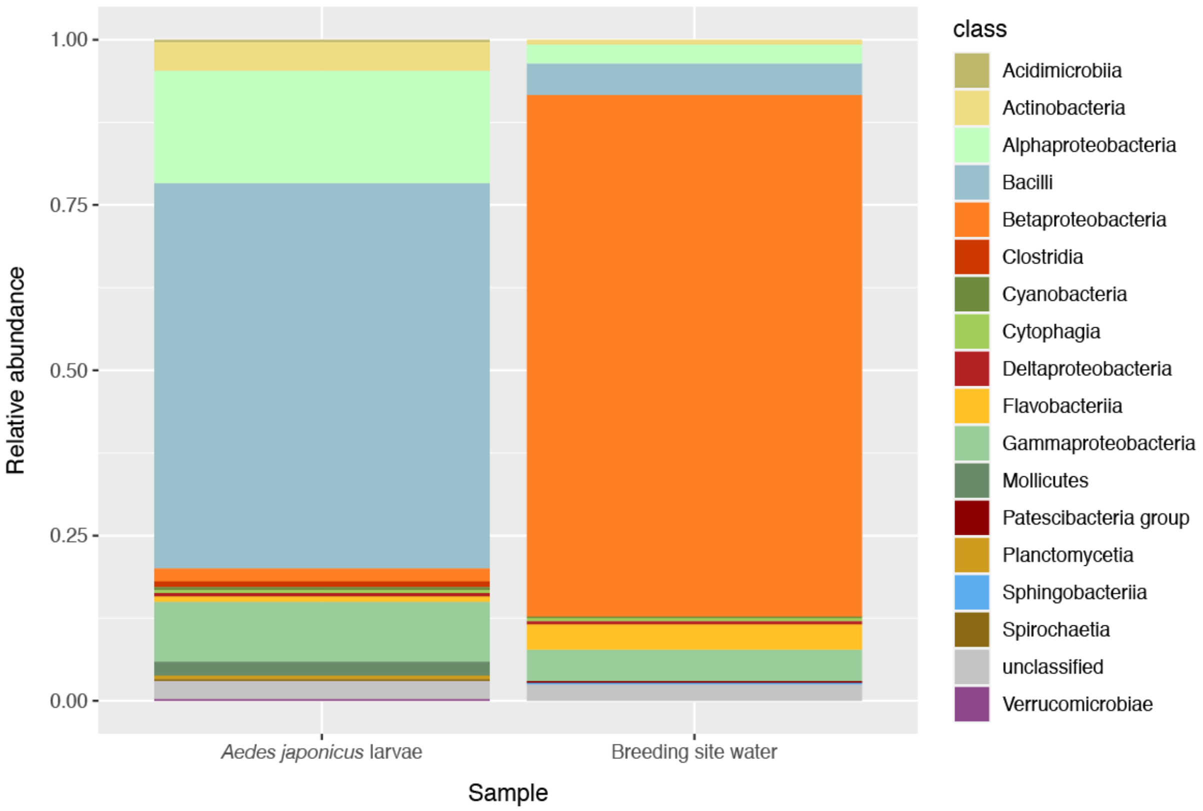

Supplementary Figure 1. Barplot of the relative abundance of bacterial classes in *Aedes japonicus* larvae and in the breeding site water. *Klebsiella* represented 1% of the Gammaproteobacteria reads in both samples. Twelve larval midguts were lysed individually overnight and pooled during DNA isolation, directly on a DNeasy Mini spin column (QIAGEN DNeasy Blood & Tissue Kit). The pooled midgut DNA sample was eluted in 80 µl AE buffer. Metagenomics library preparation and paired-end (150 bp) sequencing was performed on an Illumina NovaSeq6000 instrument by Novogene. Raw sequence reads can be found at the European Nucleotide Archive (accession number PRJEB45737). Trimmed reads were uploaded to the Kaiju web server (v1.7.3) for taxonomic classification and searched against the non-redundant database (NCBI BLAST nr+euk; last updated: 2017-05-16). Results were downloaded and stacked bar plots representing the bacterial class level were created with R (version 4.0.5) and the package ggplot2 (version 3.3.3).

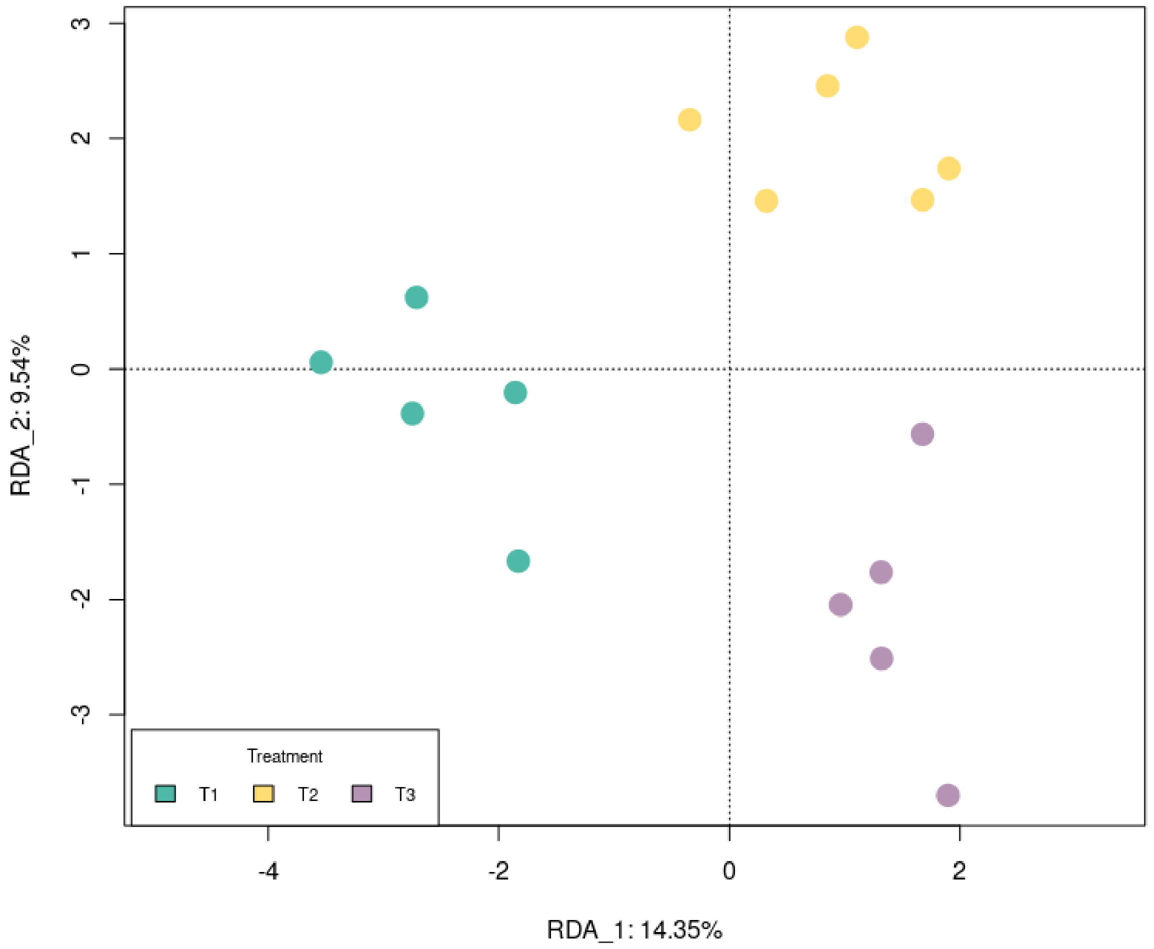

Supplementary Figure 2. Constrained ordination (RDA) showing the clustering profile among samples from each experimental treatment with regard to their affiliation. From the total variance based on Jaccard dissimilarities within the metabolite presence and absence matrix, a total of 23.89% is represented within the two constrained axes shown, distributing as follows: RDA_1(14.35%) and RDA_2(9.54%). Treatment 1 (green) included samples that served as experimental control (T_1). Treatment 2 (yellow) comprised breeding sites visited by an ovipositing female (T_2). Treatment 3 (purple) comprised breeding sites set up with manually deposited eggs (T_3).

Supplementary Table 1. Genomes used to search metabolic pathways displayed in Figure 2.

Strain	Accession No.
Acinetobacter sp. Ag1	LBMZ00000000
Acinetobacter sp. Ag2	LBNA00000000
Acinetobacter sp. AR2-3	MJIQ00000000
Asaia sp. SF2.1	AYXS00000000
Chromobacterium sp. Panama	QARX01000001
Elizabethkingia anophelis Ag1	CP007547
Elizabethkingia anophelis As1	LFKT00000000
Elizabethkingia anophelis R26	ANIW01000054
Elizabethkingia anophelis AR6-8/AR4-6	CP023403/CP023404
Enterobacter sp. Ag1	AKXM00000000
Klebsiella sp. MC1F	JAGTYC000000000
Leucobacter sp. Ag1	LAYO00000000
Lysinibacillus sp. AR18-8	MDGU00000000
Microbacterium sp. Ag1	LBCR00000000
Microbacterium sp. AR7-10	MJIR00000000
Pantoea sp. Ae16	MDJQ00000000
Pseudomonas sp. Ag1	AKVH01000000
Pseudomonas stutzeri AR9-4	MDGV00000000
Serratia fonticola AeS1	MDJO00000000
Serratia sp. Ag1	JQEI00000000
Serratia sp. Ag2	JQEJ00000000
Serratia marcescens strain Ano1	MJVB00000000
Serratia marcescens strain Ano2	MJVC00000000
Serratia marcescens As1	CP010584
Sphingobacterium sp. Ag1	LBGU00000000
Sphingomonas sp. Ag1	LAZX00000000
Staphylococcus equorum AR8-13	MDJP00000000
Staphylococcus hominis As1	LFKQ00000000
Staphylococcus hominis As2	LFKR00000000
Staphylococcus hominis As3	LFKS00000000
Stenotrophomonas maltophilia As1	LFKU00000000.
Thorsellia anophelis DSM 18579	FOHV01000000

Supplementary Table 2. Quantification of metabolites from each sample in the network displayed in Figure 3. Also shown are the m/z ratios and retention times of each metabolite. The network and associated metadata can be accessed at https://gnps.ucsd.edu/ProteoSAFe/status.jsp?task=47c0d4e60d3d41c7ac9e738004711e55

Supplementary Table 3. Pairwise PERMANOVA assessing if the variance captured by the Jaccard dissimilarities among samples from each treatment were significant and what is the effect size (R^2) that could be attributed to the conditions (biotic or abiotic) represented by each experimental group we proposed as a decoupling of the oviposition and larval development events. Treatment 1 included samples that served as experimental control. Treatment 2 comprised breeding sites visited by an ovipositing female. Treatment 3 comprised breeding sites set up with manually deposited eggs.

	Treatment 2	Treatment 3
Treatment 1	0.2464 **	0.3007 **
Treatment 2	--	0.2120 **

Signif. codes: 0 ‘***’ 0.001 ‘**’ 0.01 ‘*’ 0.05 ‘.’ 0.1 ‘ ’ 1
